# Passage-attenuated Powassan virus LI9P protects mice from lethal LI9 challenge and links envelope residue D308 to neurovirulence

**DOI:** 10.1128/mbio.00065-25

**Published:** 2025-02-25

**Authors:** Grace E. Himmler, Megan C. Mladinich, Jonas N. Conde, Elena E. Gorbunova, Marissa R. Lindner, Hwan Keun Kim, Erich R. Mackow

**Affiliations:** 1 Department of Microbiology and Immunology, Center for Infectious Disease, Renaissance School of Medicine, Stony Brook University, Stony Brook, New York, USA; 2 Department of Biological Sciences, SUNY Old Westbury, Old Westbury, New York, USA; Indiana University Bloomington, Bloomington, Indiana, USA

**Keywords:** Powassan virus, flavivirus, age-dependent lethality, murine model, live-attenuated viruses, attenuation, neuroinvasion, neurovirulence

## Abstract

Powassan virus (POWV) is an emergent tick-borne flavivirus that causes lethal encephalitic disease and chronic neurologic deficits in surviving patients. POWV-LI9 is a tick-derived isolate that causes neurovirulent disease and age-dependent lethality in mice. Serial passage of VeroE6 cells infected with LI9 resulted in eight amino acid changes in a POWV strain LI9P. LI9P fails to cause neurological sequelae, or lethality in C57BL/6 mice yet elicits neutralizing POWV antibody responses and protects mice from lethal LI9 challenge. Analysis revealed that LI9, but not LI9P, is present at high levels in the CNS, suggesting that LI9P is restricted from neuroinvasion or CNS replication. LI9 and LI9P are distinguished by a D308N envelope change within a domain associated with cell attachment. We evaluated the roles of Env-Domain III residue changes in LI9 virulence and LI9P attenuation using recombinant POWVs (recPOWVs) generated by reverse genetics. Remarkably, mutating D308N in LI9 completely abolished viral lethality and neuroinvasion in 50-week-old mice, reflecting the avirulent phenotype of LI9P. Analysis of the reciprocal N308D change in LI9P only partially restored neuroinvasion and lethality to the LI9P-N308D mutant, indicating that further LI9P residue changes contribute to LI9P attenuation. Consistent with differences in neuroinvasion, we found that rapid LI9P RNA synthesis and corresponding early IFN induction may contribute to LI9P clearance. Collectively, these findings define D308 as a determinant of POWV neuroinvasion and lethality, suggest potential mechanisms for restricted LI9P CNS entry, and reveal passage-attenuated LI9P as a candidate POWV vaccine platform.

**IMPORTANCE:**

Powassan virus (POWV) infection causes a 10% lethal encephalitis, resulting in chronic neurological symptoms in half of survivors. POWV is transmitted in as short as 15 min following tick attachment, demonstrating the need for the development of POWV vaccines and therapeutics. Mechanisms of POWV neurovirulence remain to be defined to inform vaccine and therapeutic design. Cell culture passage has successfully been used to generate live-attenuated flavivirus vaccines. Accordingly, we serially passaged POWV LI9-infected VeroE6 cells and isolated an attenuated POWV strain, LI9P, that fails to cause neurologic sequelae or murine lethality. LI9P elicits neutralizing antibody responses, protects mice from a lethal WT POWV challenge, and is a potential POWV vaccine. Analysis of attenuating mutations in LI9P revealed that changing envelope residue D308N alone in LI9 prevents POWV neurovirulence and lethality in immunocompetent mice. Altogether, this study defines viral determinants of POWV pathogenesis and attenuating mutations that inform the development of live-attenuated POWV vaccines.

## INTRODUCTION

Flaviviruses are enveloped positive-strand RNA viruses transmitted by mosquitoes and ticks (1–3). Tick-borne flaviviruses, tick-borne encephalitis virus (TBEV) and Powassan virus (POWV), annually cause ~10–15,000 annual cases in Eurasia and ~40 annual cases in North America (2, 4, 5). The increased incidence of human POWV infections over the past decade is likely due to climate change-directed geographic expansion of tick populations (6–10). Serological studies indicate that reported cases are lower than actual infection rates, suggesting that many tick-borne flavivirus cases are mild, asymptomatic, or misdiagnosed (11, 12). POWVs are present in tick saliva, and following a tick bite, transmission to mammalian hosts can occur in as short as 15 min (13, 14). POWVs are neuroinvasive and cause symptoms, including fever, headaches, confusion, and paralysis (10, 15, 16). Severe POWV cases are associated with a ~10% fatality rate with 50% of survivors having long-term neurological deficits (4, 10, 17). There are no therapeutic approaches for treating tick-borne flavivirus infections, and no licensed vaccines are available for preventing POWV infection.

POWVs are divided into two genotypes, Lineages I (LB) and II (LI9), but share ~95% amino acid identity in their envelope proteins and comprise a single serotype (4, 18, 19). Common cross-reactive neutralizing antibody targets indicate that POWV infections can be prevented by a single vaccine. POWV-LB was derived from the brain of an infected child who died of encephalitis in 1958, and LB was first isolated by passage in suckling mouse brains (20). In contrast, POWV-LI9 was isolated directly from ticks in VeroE6 cells without murine passage (21). LI9 infects VeroE6 cells and non-lytically spreads cell to cell, forming infected cell foci with or without presence of POWV-neutralizing antibodies (22). LI9 productively infects human brain microvascular endothelial cells (hBMECs) with a subset of hBMECs remaining persistently infected 6–9 dpi (21). In a transwell BBB model, LI9 is basolaterally released from hBMECs, and this suggests a potential mechanism for POWV to cross the blood–brain barrier (BBB) *in vivo* (21).

Inoculation of C57BL/6 mice with LI9 results in age-dependent lethality with a 93% survival rate in 10-week-old mice and only 18% survival in 50-week-old mice (23). Viral RNA levels in the brains of infected mice at 10 dpi are comparable between age groups; however, high POWV RNA loads at 15 dpi are sustained in the brains of 50-week-old mice but not 10-week old mice. Although, prolonged POWV loads in the CNS are linked to lower survival rates, reactive microgliosis and spongiform encephalopathy are found in both 10- and 50-week-old mice 5–15 dpi and, consistent with long term POWV CNS sequelae, in surviving mice 30 dpi (23). The age-dependent murine model of LI9 lethality permits analysis of POWV neuroinvasive mechanisms, determinants of pathogenesis, and the evaluation of potential POWV vaccines.

The lack of POWV countermeasures, combined with the severity and long term effects of POWV infection, demonstrates a need for developing POWV vaccines. The most successful flavivirus vaccine is the Yellow Fever virus (YFV) 17D vaccine (24, 25). The YFV-17D vaccine was developed by attenuating the WT Asibi strain of YFV during ~240 passages in mouse and chicken tissues and cell lines (26–29). During passage, the YFV-17D virus lost liver tropism, failed to replicate in CNS tissues, and differed from Asibi by just 32 residues, yet provides life-long immunity to WT YFV infections (30, 31). To investigate the potential for developing a live-attenuated POWV vaccine, we persistently passaged LI9-infected VeroE6 cells and evaluated progeny for mutations and an attenuating phenotype.

LI9 non-lytically infects VeroE6 cells, and this enabled us to persistently passage LI9-infected VeroE6 cells (21). Infected cells were split at ~90% confluence for ~4 months, and the resulting virus, termed LI9P, was clonally isolated, sequenced, and found to encode eight amino acid changes distributed throughout the POWV polyprotein. Importantly, subcutaneous inoculation of LI9P into 50-week-old C57BL/6 mice, failed to cause weight loss or lethality and protected mice from a subsequent challenge with a lethal dose of WT LI9 virus. Sequencing of LI9P revealed one residue change, D308N, in envelope domain III (EDIII), which is associated with cell attachment and flavivirus attenuation (32–35). In TBEV, a putative salt bridge between Env residues D308 and K311 was hypothesized to stabilize the Env protein and, when mutated, reduce viral virulence (33). Conservation of EDIII residues 308/311 in POWV provided a rationale for analyzing their role in POWV pathogenesis. To accomplish this, we used reverse genetics to mutate LI9 and LI9P EDIII residues and evaluated their role in POWV attenuation and lethality *in vivo*. Notably, we found that mutating LI9-D308N alone completely abolished viral lethality, neurologic symptoms, and neuroinvasion, mimicking attenuated LI9P infection of 50-week-old mice. Reciprocal N308D in a LI9P background resulted in partial restoration of viral lethality (10%), suggesting that additional LI9P residue differences contribute to attenuation. Collectively, we have generated a passage-attenuated POWV, LI9P, that fails to cause neurologic symptoms, elicits neutralizing antibody responses, and protects mice from a lethal WT LI9 challenge. These findings provide a potential backbone for developing live-attenuated POWV vaccines and identify Env D308 as a determinant of POWV neurovirulence.

## RESULTS

### Generation of persistently passaged POWV strain LI9P

**Fig 1:**
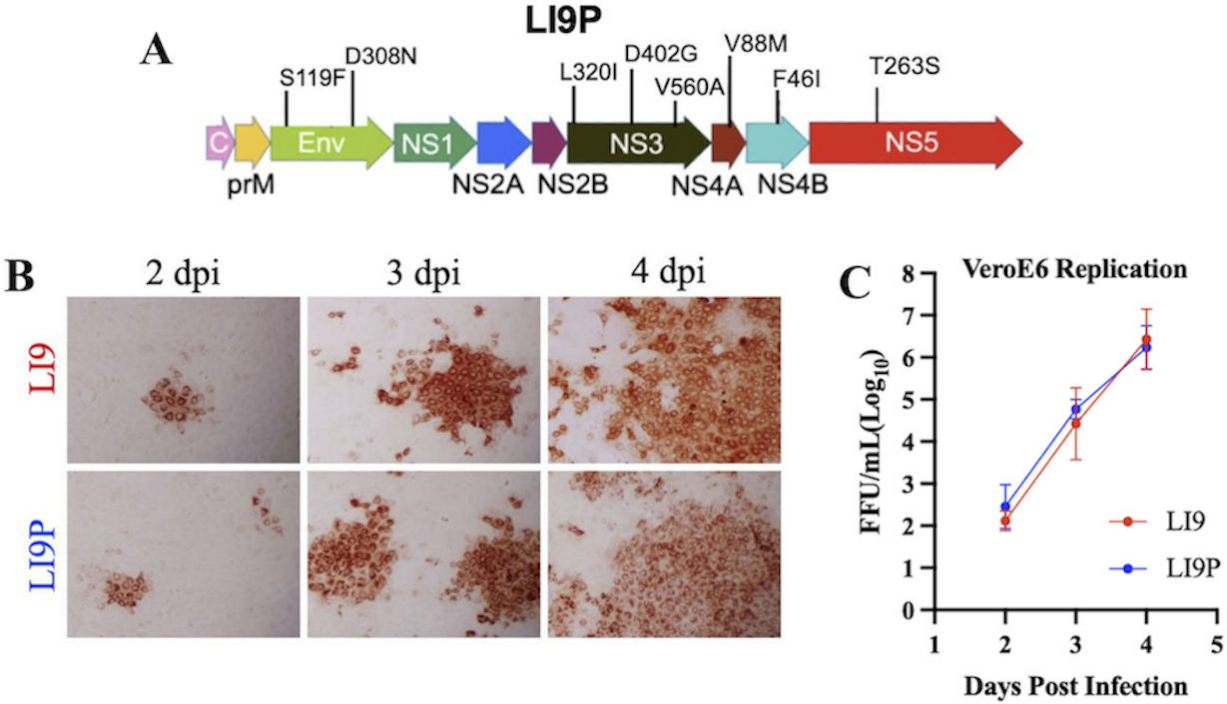
Generation of persistently passaged POWV strain LI9P. (**A**) Amino acid differences between LI9 and LI9P determined by sequence analysis and presented relative to their position in viral proteins. (**B**) VeroE6 cells were infected with LI9 or LI9P at an MOI of 0.01, cells were methanol fixed at 2, 3, and 4 dpi and immunoperoxidase stained for POWV antigen. (**C**) Supernatants collected from VeroE6 cells infected with LI9 or LI9P at an MOI of 0.01 at 2, 3, and 4 dpi then titered on VeroE6 cells.

LI9 is a tick-derived POWV strain that is neurovirulent and causes age-dependent lethality in C57BL/6 mice (23). In VeroE6 cells, LI9 spreads non-lytically and cell to cell even in the presence of neutralizing antibodies (22). The absence of *in vitro* cytopathology, and the success of passage attenuated flavivirus vaccines, prompted us to persistently passage VeroE6 cells infected with LI9 and evaluate potentially attenuating mutations in POWV progeny. To accomplish this, we continuously passaged LI9-infected VeroE6 cells for ~4 months (~24 passages). Viral stocks were cloned by end-point dilution, and a POWV isolate termed “LI9P” was sequenced. While no changes in the 5′ or 3′ UTRs were observed, we found two silent mutations and eight stably maintained amino acid differences from parental LI9. LI9P contains two nucleotide changes (T120C and T789C), two residue changes in the envelope protein, three changes in NS3, and one change each in NS4A, NS4B, and NS5 (Fig. 1A). A comparison of LI9 and LI9P demonstrates that both POWVs spread similarly in VeroE6 cells, initially forming infected cell foci 2–3 dpi with subsequent viral dissemination throughout the monolayer (Fig. 1B). LI9 and LI9P infection of VeroE6 cells resulted in nearly identical rates of replication and supernatant titers 2–4 dpi (Fig. 1C). Collectively, these findings demonstrate that mutations in LI9P do not significantly alter replication in VeroE6 cells.

### POWV LI9P is attenuated in C57BL/6 mice

**Fig 2:**
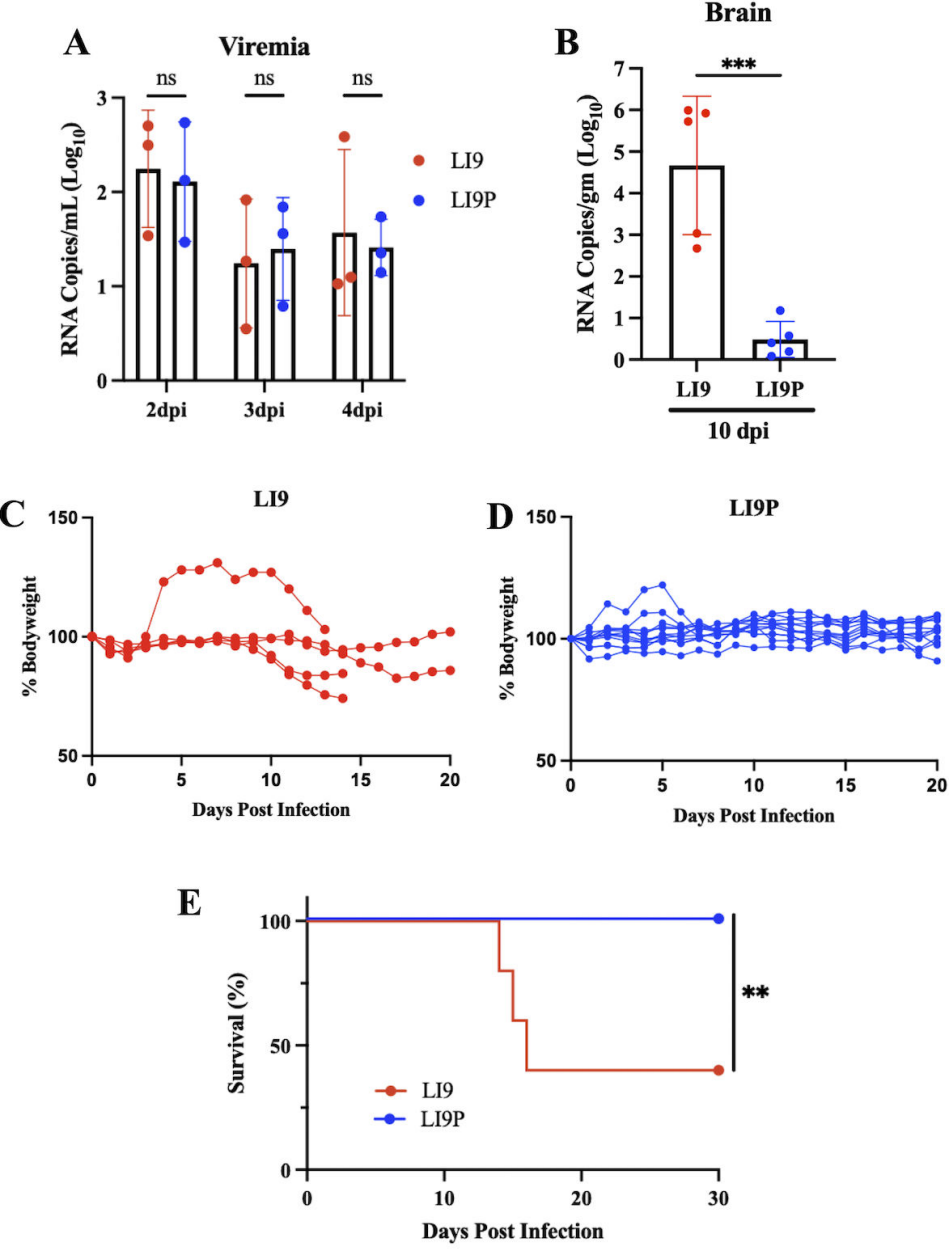
POWV LI9P is attenuated in C57BL/6 mice. (**A**) The 50-week-old C57BL/6 mice were s.c. infected with 2,000 FFU of LI9 or LI9P. Mice were bled at 2, 3, and 4 dpi by cardiac puncture, serum isolated, and viremia determined by qRT-PCR analysis of NS5 containing transcripts normalized to control sera. Each time point consists of *n* = 3 per POWV strain with data shown as means ± SD and analyzed for significance using two-way ANOVA. (**B**) The 50-week-old C57BL/6 mice were s.c. infected with 2,000 FFU of LI9 or LI9P. Brains (*n* = 5 per POWV strain) were collected at 10 dpi, and viral load was determined via qRT-PCR and normalized to negative control brains. Data shown as means ± SD and analyzed for statistical significance with unpaired *t*-test (***P < 0.001). (**C**) Individual mouse body weights as percent of body weight at 0 dpi from 50-week-old C57BL/6 mice s.c. infected with 2,000 FFU of LI9 (*n* = 5) or (**D**) LI9P (*n* = 10). (**E**) Kaplan–Meier curves of POWV infection following s.c. infection of 2,000 FFU of LI9 (*n* = 5) or LI9P (*n* = 10) analyzed for significance by log-rank test (**P < 0.01).

LI9 inoculation into immunocompetent C57BL/6 mice results in virulent disease associated with neuroinflammation, and lethality that increases from 7.2% in 10-week-old mice to 82% in 50-week-old mice (23). Although LI9 and LI9P replicate similarly in VeroE6 cells, mutations in LI9P were acquired in the absence of IFN selective pressure and may confer attenuation in innate immune competent settings. To evaluate LI9P virulence, we s.c. inoculated 50-week-old mice with LI9 or LI9P (2,000 FFU) and assayed viral load, spread and lethality *in vivo*. We found comparable viremia in both LI9 and LI9P-infected mice 2–4 dpi (Fig. 2A). In contrast, brain viral loads in LI9 or LI9P s.c. infected 50-week-old mice differed dramatically at 10 dpi. While LI9 brain RNA levels reach 10^4.5^ copies/gm at 10 dpi, LI9P brain RNA levels were at or just above the limit of RNA detection by qRT-PCR (Fig. 2B). Consistent with high LI9 viral loads and minimal LI9P transcripts in the CNS of 50-week-old mice, we found that LI9 induced high levels of IFN-β, IL-12, CCL2, TNF-α, CXCL10, and IL-6 RNAs, while these transcripts were at background levels in the brains of LI9P-infected mice (Fig. S1). These findings suggest that LI9P either fails to enter the CNS or is replication restricted within the CNS. As previously reported for LI9 infected 50-week-old mice (23), decreases in body weight began 10 dpi (Fig. 2C), with onset of neurologic symptoms (hindlimb paralysis, ataxia, and weak grip) beginning 11–13 dpi and lethality (60%, *n* = 5) 13–15 dpi (Fig. 2E). Unexpectedly, one mouse gained weight between 3 and 4 dpi yet exhibited similar weight loss and disease onset kinetics as other LI9-infected mice. In contrast to LI9-infected mice, LI9P-infected mice (*n* = 10) failed to lose weight (Fig. 2D) or show neurologic symptoms and had a 100% survival rate (Fig. 2E). These findings suggest that LI9P is attenuated and lacks the ability to spread to the CNS or replicate in the brain.

### LI9P protects mice from lethal LI9 challenge

**Fig 3:**
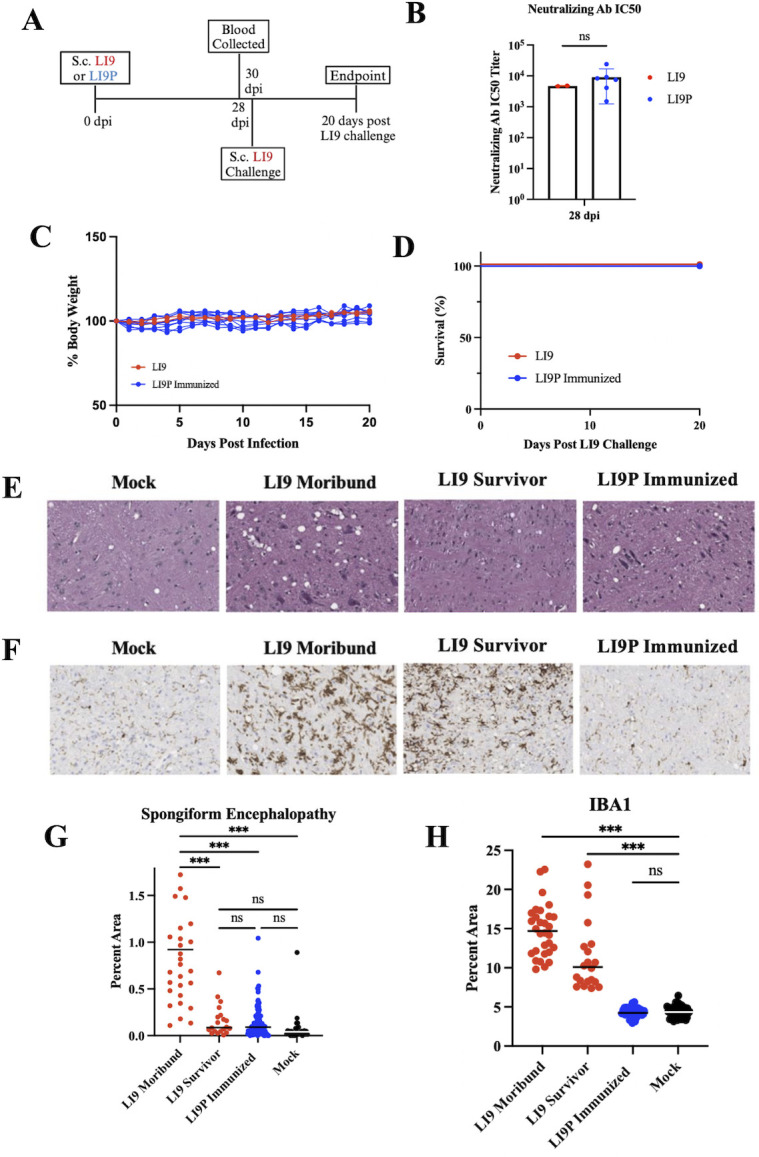
LI9P protects mice from lethal LI9 challenge. (**A**) Experimental design schematic: 50-week-old C57BL/6 mice were s.c. infected with 2,000 FFU of LI9 (*n* = 5) or (**D**) LI9P (*n* = 10). Survivors of infection were bled retro-orbitally at 28 dpi and challenged with a lethal dose of LI9 (2,000 FFU s.c.) at 30 dpi. Changes in body weight, neurological symptoms, and lethality were monitored for 20 days post-LI9 challenge. (**B**) Sera neutralizing antibody titers (IC50) from surviving LI9- (*n* = 2) or LI9P-infected mice (*n* = 10) were analyzed. Data are presented as the means ± SD and analyzed with unpaired *t*-test. (**C-D**) At 30 dpi, C57BL/6 mice surviving prior LI9 or LI9P infection were challenged with 2,000 FFU LI9 and monitored for (**C**) body weight changes and survival. (**D**) Kaplan–Meier curves for 20 days post-LI9 challenge. (**E**) Representative images of H&E-stained midbrains from LI9-, LI9P-, or mock-infected 50-week-old mice. LI9-infected brains from moribund (terminal stage of infection) or mice surviving LI9 infection are presented. (**F**) Representative images of IBA1 IHC-stained midbrains from LI9-, LI9P-, or mock-infected 50-week-old mice. (**G**) ImageJ quantification of spongiform encephalopathy lesions (percent area of 10 sections each) for mock (*n* = 2), LI9 moribund (*n* = 3), LI9 survivors (*n* = 2), and LI9P infected brains (*n* = 10) shown in Fig. 3E. Bars indicate mean and statistical significance determined using one-way ANOVA. (**H**) ImageJ quantification of IBA1 IHC staining from 10 brain sections for each mock (*n* = 2), LI9 moribund (*n* = 3), LI9 survivors (*n* = 2), and LI9P- infected mice (*n* = 5). Bars indicate mean and statistical significance determined using one-way ANOVA (***P < 0.0001).

To assess LI9P attenuation and potential as a vaccine candidate, we subcutaneously inoculated 50-week-old C57BL/6 mice with 2,000 FFU of LI9P (Fig. 2C trough E) collected blood retro-orbitally 28 dpi and challenged the surviving mice with a lethal dose of LI9 (Fig. 3A). Similar titers of neutralizing antibodies were detected in sera 28 dpi following infection with LI9 or LI9P (Fig. 3B). At 30 dpi, mice surviving LI9P (10/10) or LI9 (2/5) infection were challenged with a lethal dose of LI9 (2,000 FFU). In all mice previously infected with LI9 or LI9P, a LI9 challenge failed to cause body weight loss (Fig. 3C) or neurologic symptoms and resulted in 100% survival (Fig. 3D). These findings indicate that LI9P infection elicits neutralizing antibody responses and protects mice from lethal LI9 infection. To evaluate neuroprotective effects of LI9P infection, we evaluated brains from LI9P-infected, and subsequently LI9-challenged, mice for spongiform encephalopathy and microglia activation. Hematoxylin and eosin (H&E) staining of brains from LI9 infected 50-week-old mice collected in a moribund state (13–15 dpi) reveals severe spongiform encephalopathic lesions in the midbrain (Fig. 3E) that are significantly reduced in mock, LI9 survivors, and LI9P immunized/LI9-challenged brains (Fig. 3G). We found that brains from LI9-infected moribund mice and LI9 survivors that were rechallenged with LI9 have high levels of IBA1 staining (Fig. 3F and H), indicative of microglial activation directed by LI9 infection. In contrast, the brains of LI9P immunized mice failed to show elevated IBA1 staining following LI9 challenge (Fig. 3F and H). Collectively, these findings indicate that LI9P is attenuated in 50-week-old C57BL/6 mice and directs neuroprotective responses that inhibit neuroinvasion and inflammatory microglial activation in mice challenged with lethal LI9.

### POWV LI9 is attenuated by a single D308N mutation

**Fig 4:**
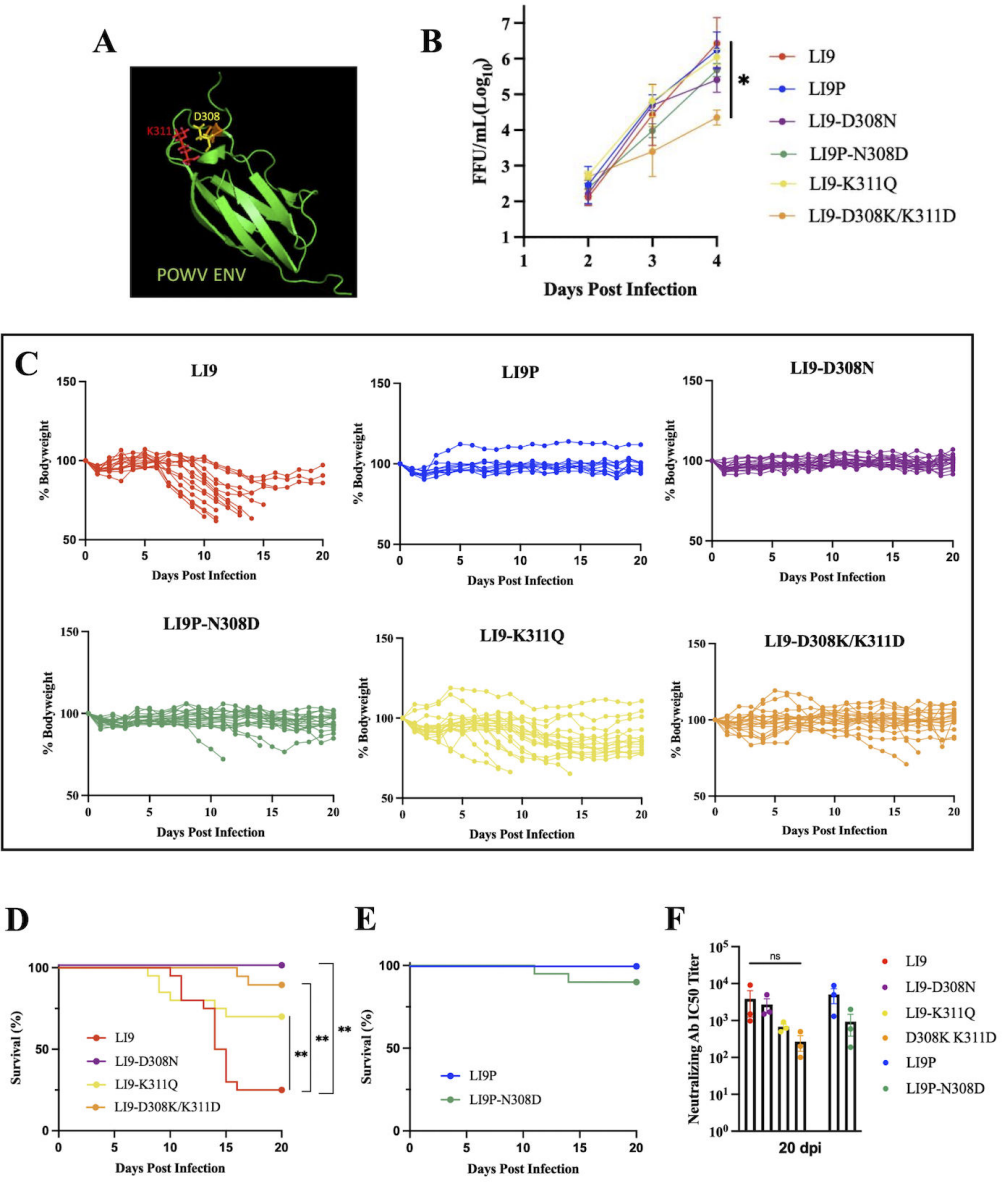
POWV LI9 is attenuated by a single D308N mutation. (**A**) Pymol model of POWV Env (PDB accession number 7SGT.PDB) highlighting putative salt bridge interaction between residues D308 and K311. (**B**) Replication kinetics of CPER generated recPOWVs in VeroE6 cells. Supernatants from VeroE6 cells infected with LI9, LI9P, LI9-D308N, LI9P-N308D, LI9-K311Q, or LI9-D308K/K311D at an MOI of 0.01 were collected at 2–4 dpi then titered on VeroE6 cells. Data presented as means ± SD analyzed using two-way ANOVA (*P < 0.05). (**C**) Fifty-week-old C57BL/6 mice were s.c. infected with 2,000 FFU of LI9 (*n* = 15), LI9P (*n* = 10), LI9-D308N (*n* = 20), LI9P-N308D (*n* = 20), LI9-K311Q (*n* = 20), or LI9-D308K/K311D (*n* = 20) and kinetically monitored for individual body weight changes. Data presented as a percent of body weight before infection (0 dpi). (**D-E**) Kaplan–Meier curves of 50-week-old C57BL/6 mice s.c. infected with 2,000 FFU of (**D**) LI9 (*n* = 15), LI9-D308N (*n* = 20), LI9-K311Q (*n* = 20), LI9-D308K/K311D (*n* = 20), (**E**) LI9P (*n* = 10), and LI9P-N308D (*n* = 20) analyzed for significance by log-rank test (**P < 0.01). (**F**) Sera neutralizing antibody titers (IC50) from infected mice 20 dpi were analyzed. Data are presented as the means ± SEM and analyzed with one-way ANOVA.

Envelope domain III (EDIII) residues are conserved in POWV and TBEV; in other flaviviruses, this domain has been associated with flavivirus cell attachment and attenuation (32–34, 36). In TBEV, EDIII D308 forms a putative salt bridge with K311 that may contribute to cell attachment and partial attenuation (33). One EDIII residue differs between LI9 and LI9P Env proteins, D308N (Fig. 4A). To evaluate roles for D308 and K311 in LI9 virulence and attenuation, we generated recPOWV mutants using a CPER reverse genetics system. CPER fragments containing Env sequences from LI9 or LI9P viruses were amplified and used to generate single EDIII mutant viruses recLI9-D308N and recLI9P-N308D. Additional LI9 Env residue K311 changes (K311Q; K311A; K311F) were evaluated for their ability to disrupt potential salt bridge interactions. Although K311A and K311F mutations failed to produce viable recLI9 mutants, a virus containing K311Q was stably produced along with a double mutant, recLI9-D308K/K311D, that inverts putative salt bridge residues. CPER-generated recPOWVs stocks were grown, sequence verified, and comparatively analyzed for recPOWV replication in VeroE6 cells 2–4 dpi. We found that recLI9-D308N, recLI9-N308D, and recLI9-K311Q replicate at similar rates and produce similar viral titers as parental LI9 and LI9P strains. In contrast, the recLI9-D308K/K311D replication rate is reduced, producing 2-log lower infectious titers at 4 dpi (Fig. 4B).

To assess the lethality of recPOWV mutants, 50-week-old C57BL/6 mice were s.c. infected with 2,000 FFU of each recPOWV and monitored for body weight changes and survival. As previously reported, LI9 infection of 50-week-old mice results in severe body weight loss (Fig. 4C), neurologic symptoms, and 80% lethality (12/15) (Fig. 4D). Replicating data in Fig. 2E, we found that LI9P infection of 50-week-old mice (*n* = 10) failed to cause weight loss (Fig. 4C), neurologic symptoms, or lethality (Fig. 4E). Remarkably, we found that infection of 50-week-old mice with the recLI9-D308N mutant, containing a single EDIII change, failed to cause changes in body weight, neurological symptoms, or lethality (*n* = 20) (Fig. 4C and D). This finding demonstrates a clear role for Env residue D308 in POWV LI9 virulence and the attenuation of LI9P. Furthermore, when 50-week-old mice previously infected with recLI9-D308N are challenged with a lethal dose of LI9, the mice are fully protected from LI9-directed weight loss, neurologic symptoms, and lethality (*n* = 9) (Fig. S2A and B). In contrast, the reciprocal change (N308D) in the recLI9P-N308D virus did not fully restore lethality in 50-week-old mice, as 3/20 mice exhibited severe weight loss and neurologic symptoms, with lethality in 2/20 mice (Fig. 4C and E). Findings that 17/20 mice failed to lose weight, show neurologic symptoms, or succumb to recLI9P-N308D infection suggest that additional residue changes contribute to LI9P attenuation.

Other changes in residues adjacent to EDIII D308 also altered LI9 virulence, with or without mutating D308. Although inoculating 50-week-old mice with recLI9-K311Q caused weight loss (Fig. 4C) and neurologic symptoms in all mice (*n* = 20), it was only 20% lethal and, as a result, significantly attenuated compared with LI9 infection (80% lethal) (Fig. 4D). Infection of mice with the double mutant, recLI9-D308K/K311D, resulted in body weight loss, neurologic symptoms, and lethality in 10% of mice (2/20) (Fig. 4C and D). Despite attenuation differences, 100% of mice surviving infection by POWV mutants seroconverted and elicited POWV-specific neutralizing antibodies (Fig. 4F). These data indicate that modifying WT POWV LI9 Env 308/311 residues alters neurovirulence and lethality *in vivo* while delineating fundamental viral EDIII residues as determinants of POWV virulence and attenuation.

### POWV lethality correlates with increased viral load in the spleen

**Fig 5:**
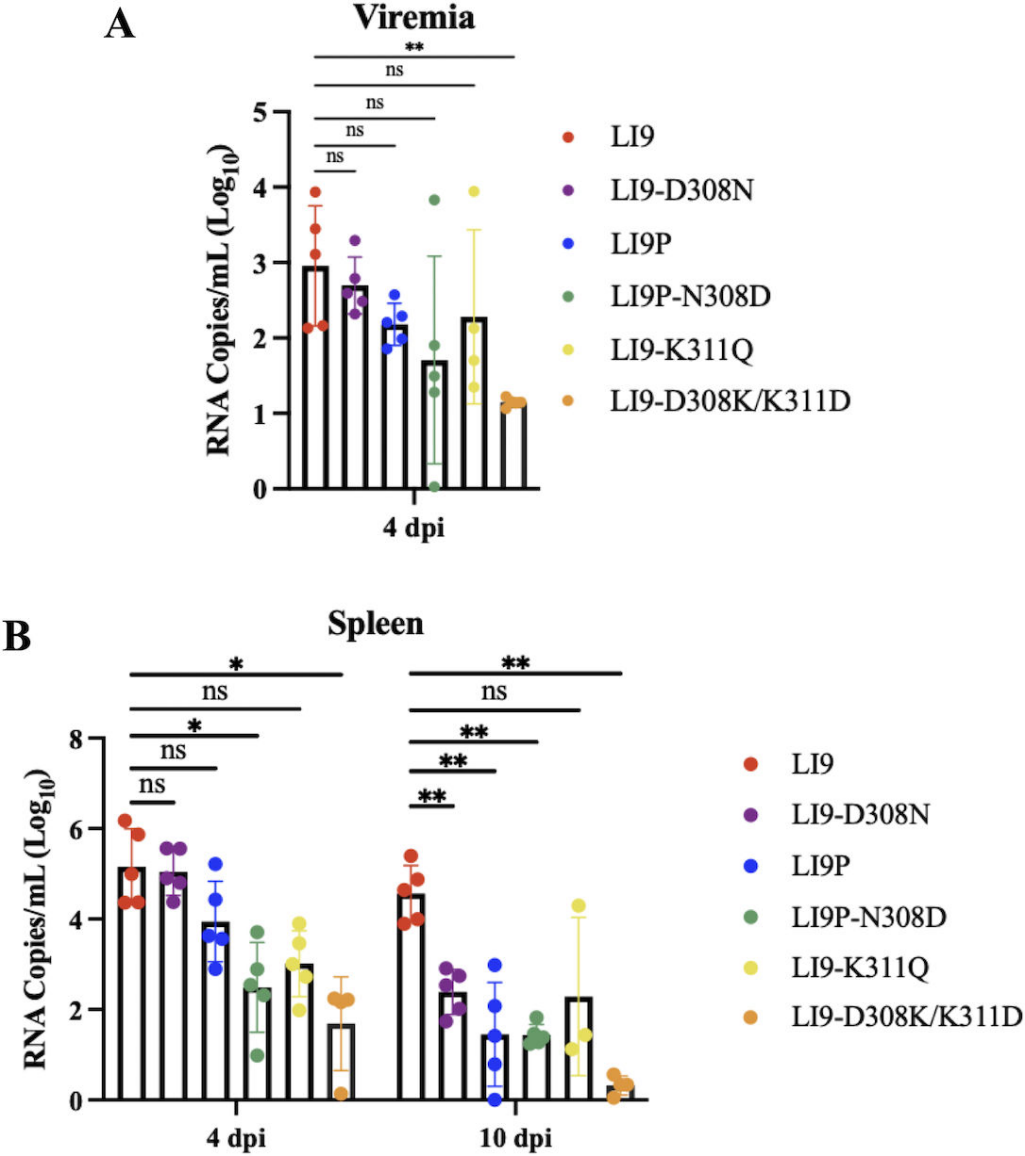
POWV lethality correlates with increased viral load in the spleen. (**A**) Ten-week-old C57BL/6 mice were s.c. infected with 2,000 FFU of LI9 (*n* = 5), LI9P (*n* = 5), LI9-D308N (*n* = 5), LI9P-N308D (*n* = 5), LI9-K311Q (*n* = 5), or LI9-D308K/K311D (*n* = 5). Mice were bled at 4 dpi by cardiac puncture and viremia determined by qRT-PCR normalized to negative controls. Data presented as means ± SD analyzed using one-way ANOVA (*P < 0.05; **P < 0.01). (**B**) Ten-week-old C57BL/6 mice were s.c. infected with 2,000 FFU of LI9 (*n* = 5), LI9P (*n* = 5), LI9-D308N (*n* = 5), LI9P-N308D (*n* = 5), LI9-K311Q (*n* = 5), or LI9-D308K/K311D (*n* = 5). Spleens were collected at 4 and 10 dpi, and RNA viral load was determined by qRT-PCR normalized to negative controls. Data presented as means ± SD analyzed using two-way ANOVA (*P < 0.05; **P < 0.01).

We evaluated the relative levels of LI9, LI9P, and recPOWVs in the sera and spleens of s.c. inoculated (2,000 FFU) 10-week-old C57BL/6 mice. In the sera of LI9-infected mice, we observed between 10^1^–10^3^ copies of POWV RNA/mL 4 dpi (Fig. 5A). Viral loads in the sera of mice infected with LI9P, recLI9-D308N, recLI9P-N308D, and recLI9-K311Q were not statistically different from viral load present in the sera of LI9-infected mice, indicating a common level of viremia across POWV strains. Consistent with reduced *in vitro* replication of recLI9-D308K/K311D (Fig. 4B), significantly decreased viremia was observed in infected mice (Fig. 5A). To further investigate systemic POWV dissemination, we kinetically monitored viral loads in the spleen. We found that infection with LI9, LI9P, recLI9-D308N, or recLI9-K311Q results in similar levels of viral load in the spleen at 4 dpi. In contrast, by 10 dpi, viral loads in the spleen were 2–4 logs higher in LI9-infected mice versus mice infected with fully (recLI9P and recLI9-D308N) or partially attenuated POWVs (recLI9P-N308D, recLI9-K311Q, and recLI9-D308K/K311D) (Fig. 5B). These findings suggest an association between high splenic viral loads 10 dpi and increases in murine lethality (Fig. 4D).

### Envelope D308 confers POWV LI9 neurovirulence

**Fig 6:**
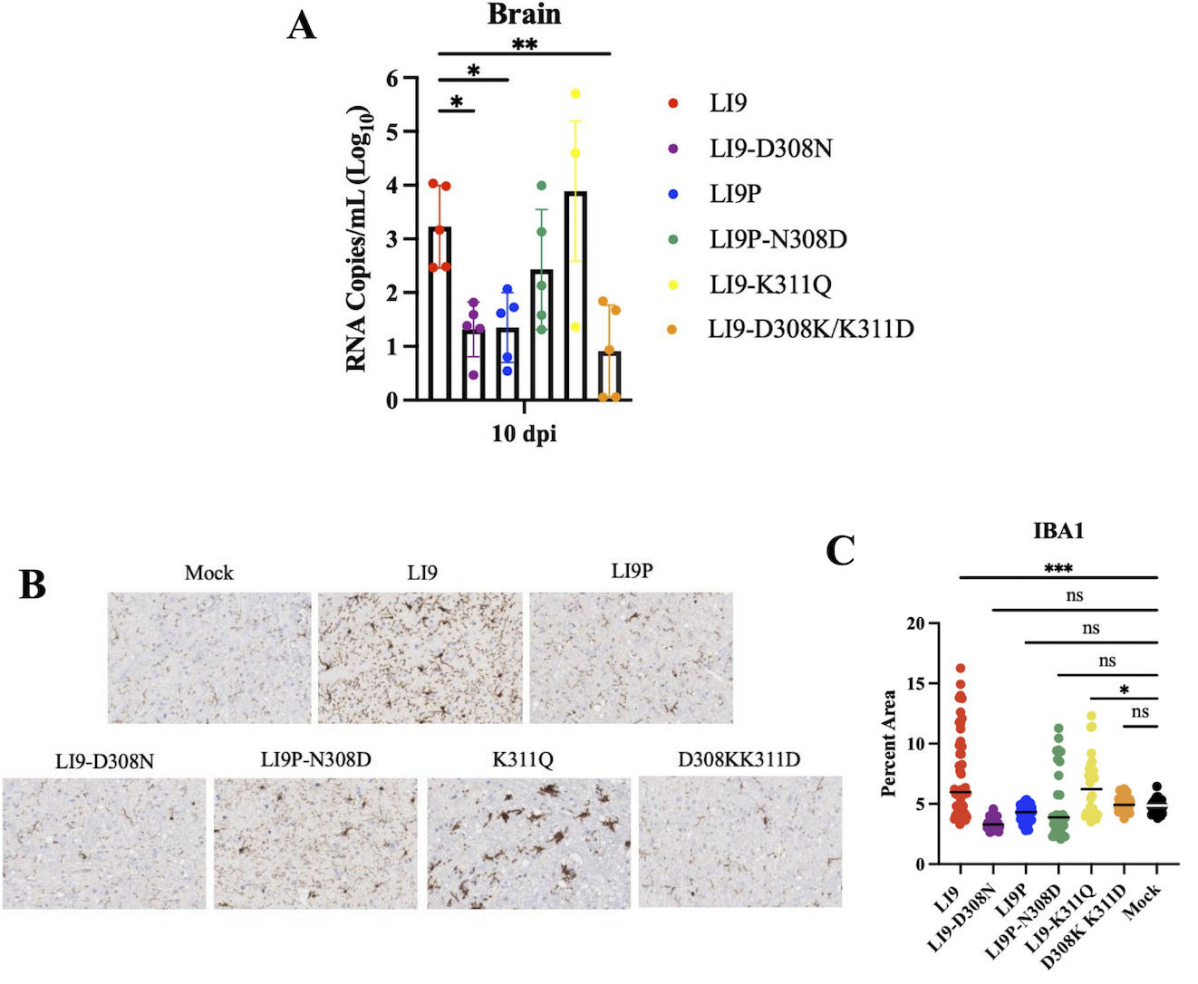
Envelope D308 confers POWV LI9 neurovirulence 10-week-old C57BL/6 mice were s.c. infected with 2,000 FFU of LI9 (*n* = 5), LI9P (*n* = 5), LI9-D308N (*n* = 5), LI9P-N308D (*n* = 5), LI9-K311Q (*n* = 5), or LI9-D308K/K311D (*n* = 5), and brains were collected at 10 dpi. (A) RNA was isolated from brains, and viral load was determined via qRT-PCR of NS5 POWV transcripts and normalized to negative controls. Data presented as means ± SD analyzed using one-way ANOVA (*P < 0.05; **P < 0.01). (B) Representative images of IBA1 IHC staining of the midbrain in LI9, LI9P, LI9-D308N, LI9P-N308D, LI9-K311Q, LI9-D308K/K311D, or mock-infected 10-week-old mice. (C) ImageJ quantification of IBA1 IHC staining of 10 brain sections each from mock (*n* = 2), LI9 (*n* = 5), LI9P (*n* = 5), LI9-D308N (*n* = 3), LI9P-N308D (*n* = 3), LI9-K311Q (*n* = 3), or LI9-D308K/K311D- infected mice (*n* = 3). Bars indicate median and statistical significance determined using one-way ANOVA (*P < 0.01; **P < 0.001, ***P < 0.0001).

While LI9 directs age-dependent lethality in C57BL/6 mice, LI9-infected mice of all ages were found to have spongiform encephalitis, microglial activation, and increased viral loads in the brain. To assess differences in neuroinvasion of recPOWVs, we subcutaneously inoculated 10-week-old mice with 2,000 FFU of each POWV and evaluated viral RNA levels in the CNS at 10 dpi. In the brains of LI9, recLI9P-N308D, and recLI9-K311Q, we detected 10^2^–10^4^ viral RNA copies/mL, compared with RNA levels of ~10 copies/gm in LI9P, recLI9-D308N, and recLI9-D308K/K311D infected brains (Fig. 6A). These findings link LI9, recLI9P-N308D, and recLI9-K311Q induced lethality to the entry and accumulation of virus in the CNS and, conversely, suggest that reduced CNS tropism or replication is associated with attenuated POWVs (LI9P, recLI9-D308N, and recLI9-D308K/K311D). To further assess mechanisms of POWV neurovirulence, we comparatively evaluated and quantified the prevalence of activated microglia (IBA1+) in brains 10 days post-POWV infection. We observed increased IBA1 staining in the brains of LI9 and recLI9-K311Q infected mice versus controls, reflecting POWV induced microglial activation (Fig. 6B and C). In contrast, reduced IBA1 staining in the brains of LI9P-, recLI9-D308N-, and recLI9-D308K/K311D-infected mice reflect findings of mock-infected controls (Fig. 6B and C). Overall, microgliosis found in the brains of LI9 and recLI9-K311Q is consistent with increased CNS viral loads, neuropathology, and increased POWV lethality. In contrast, the absence of microgliosis and high CNS viral loads in the brains of mice infected with attenuated POWVs (LI9P, recLI9-D308N) suggest parameters for differentiating neurovirulent and attenuated POWV strains.

### Differences in LI9 and LI9P dissemination in hBMECs are consistent with altered IFN responses

**Fig 7:**
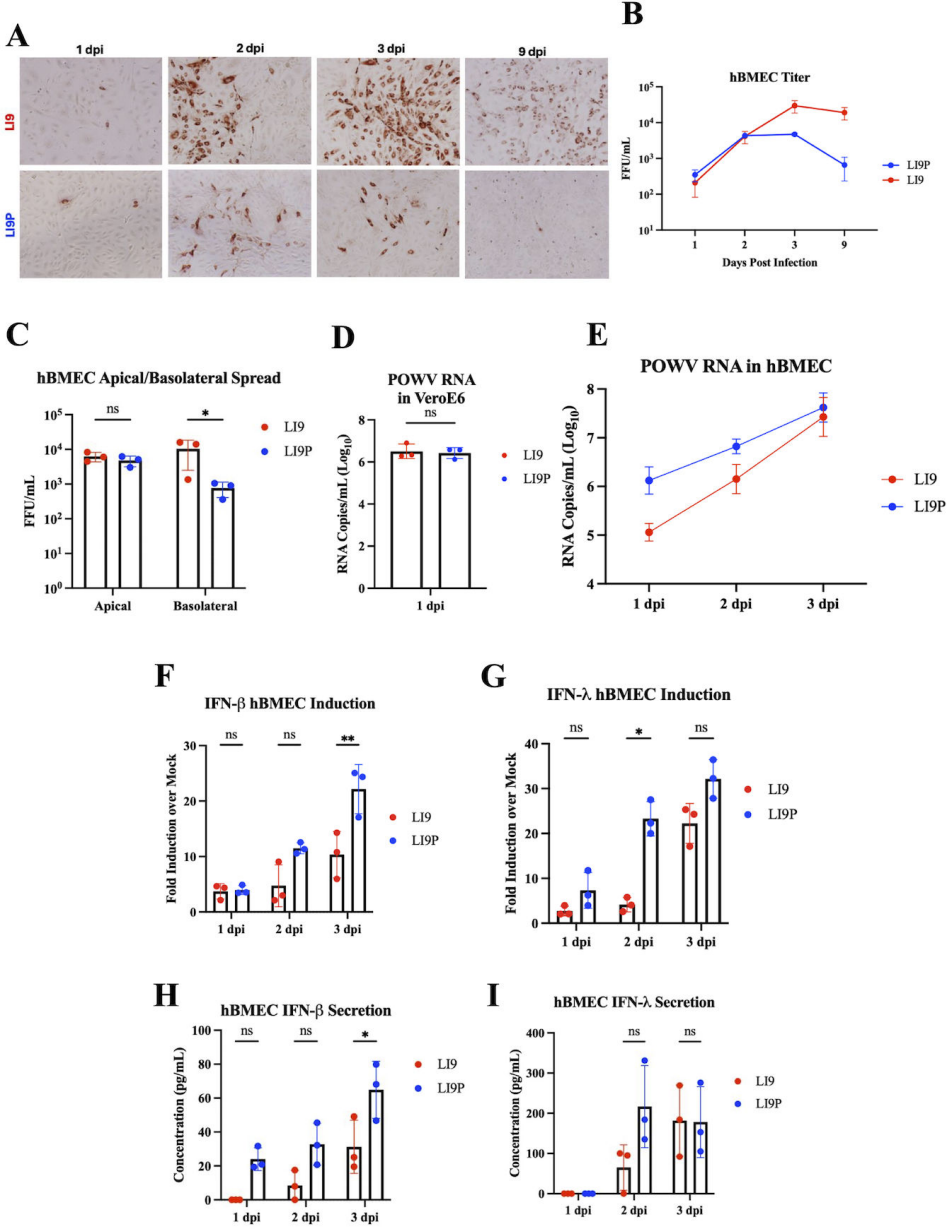
Differences in LI9 and LI9P dissemination in hBMECs are consistent with altered IFN responses. (**A**) hBMECs were infected with LI9 or LI9P at an MOI of 0.1, cells were fixed and immunoperoxidase stained at 1, 2, 3, and 9 dpi. (**B**) Supernatants were harvested at 1, 2, 3, and 9 dpi from LI9- or LI9P-infected hBMECs (MOI of 0.1) then titered on VeroE6 cells. (**C**) hBMECs grown on Transwell inserts for 7 days were evaluated for polarization by trans-endothelial resistance (TEER) and apically infected with LI9 or LI9P at a MOI of 1. At 3 dpi, supernatants were collected from apical and basolateral chambers at 3 dpi and titered on VeroE6 cells. (**D**) Following infection of LI9 and LI9P (MOI of 1), comparable RNA levels in VeroE6 cell lysates were evaluated at 1 dpi by qRT-PCR of NS5 transcripts. (**E-I**) hBMECs were LI9 and LI9P infected (MOI = 1) ,and 1–3 dpi transcriptional responses were assessed in cell lysates via qRT-PCR. (**E**) Viral replication was assessed via POWV NS5 transcripts. (**F**) IFN-β and (**G**) IFN-λ mRNAs were analyzed for induction differences relative to mock controls. Supernatants of LI9- and LI9P-infected hBMECs (MOI = 1) were assayed by ELISA (R&D systems) for (**H**) IFN-β secretion (LoD = 7.8 pg/mL) and (**I**) IFN-λ secretion (LoD = 62.5).

Previous studies indicated that *in vitro*, LI9 persistently infects primary human brain microvascular cells (hBMECs) (21), which form the blood–brain barrier (BBB) and function to protect CNS compartments. In transwell BBB models, LI9 is released both apically and basolaterally from hBMECs, which suggests a potential neuroinvasion mechanism (37–39). Here, we comparably infected hBMECs with LI9 and LI9P (MOI = 0.1) and kinetically evaluated viral spread. We found that LI9 persistently infects hBMECs and maintains LI9 titers in cell supernatants out to 9 dpi (Fig. 7A and B). Although LI9P infects hBMECs 1–3 dpi, replication and spread are restricted in hBMECs, with nearly complete POWV antigen clearance from hBMEC monolayers by 9 dpi (Fig. 7A and B). Polarized hBMECs on transwell inserts were infected with LI9 or LI9P (MOI = 1) and analyzed apical and basolateral POWV release at 3 dpi. We found that LI9 and LI9P are released at similar levels from the apical side of hBMECs, while ~10-fold more infectious LI9 was released basolaterally than LI9P (Fig. 7C). These findings are distinguished from nearly identical LI9 and LI9P replication rates and titers in interferon-deficient VeroE6 cells (Fig. 1C) and comparable RNA synthesis levels within VeroE6 cells (Fig. 7D). In contrast, following comparable LI9 and LI9P infection of hBMECs (MOI = 1), cellular RNA copies of LI9P are ~1 log higher than LI9 at 1 dpi (Fig. 7E) and accumulate at a reduced rate from 1 to 3 dpi compared with LI9 (Fig. 7E).

To determine if differences in RNA levels and replication within hBMECs were indicative of altered IFN responses, we compared LI9- and LI9P-directed IFN-β and IFN-λ induction. Analysis of the replication of LI9, LI9P, and reciprocal D308N and N308D mutants in hBMECs revealed higher viral RNA levels in cells 1 dpi following infection by attenuated strains LI9P and recLI9-D308N compared with highly virulent LI9 and partially virulent LI9P-N308D (Fig. S3A). Comparison of IFN induction demonstrated that LI9P induced IFN-β transcripts approximately twofold higher than LI9 or either D308 mutant 2–3 dpi and IFN-λ ~2–4-fold higher than LI9 or D308 mutants 2 dpi (Fig. S2B and C). In LI9P-infected hBMECs, we observed higher IFN-β and IFN-λ induction 2–3 dpi compared with LI9 infection (Fig. 7F and G), and at 3 dpi significantly higher levels of secreted IFN-β were present in the supernatants of LI9P versus LI9-infected hBMECs (Fig. 7H and I). Enhanced IFN responses of LI9P over LI9 are likely to be directed by passage-acquired LI9P mutations in NS3 (3 AAs), NS4A (1 AA), NS4B (1 AA) ,and NS5 (1 AA) proteins during persistent replication in IFN-deficient VeroE6 cells. However, the recLI9P-N308D mutant IFN responses were indistinguishable from attenuated LI9-D308N (Fig. S3B and C), suggesting that the presence of D308 itself plays a role in both reduced replication efficiency and elicited IFN responses in POWV-infected hBMECs.

## DISCUSSION

In this study, we generated a passage-attenuated POWV and defined viral determinants of neurovirulence within domain III of the envelope protein. EDIII has previously been associated with cell attachment, clearance, attenuation, and neurovirulence by discrete flaviviruses (33, 35, 40–42); however, specific binding interactions and attenuation mechanisms conferred by changes in this domain remain to be determined. Studies of POWV virulence have been hampered by the lack of a robust animal disease model and the inability to generate recombinant POWV mutants. Here, we use a POWV murine model that results in highly lethal neurovirulent disease in immunocompetent mice, develop a passage-attenuated POWV, and use CPER reverse genetics to evaluate the role of specific Env mutations in viral virulence and attenuation. Our findings define determinants of POWV neurovirulence within EDIII residues and develop a live-attenuated vaccine candidate, LI9P, that prevents POWV neuroinvasion and lethality in a mouse model.

Persistent passage of LI9 in tissue culture is facilitated by the absence of VeroE6 cell cytopathology. After 4 months, ~24 passages, we clonally isolated and sequenced POWV-LI9P and found eight amino acid substitutions in the polyprotein that differed from LI9. LI9P virulence was evaluated in 50-week-old C57BL/6 mice that were recently found to be a robust POWV murine model, resulting in weight loss, neurologic symptoms, and ~80% lethality following LI9 inoculation (23). However, following LI9P infection, 50-week-old C57BL/6 mice failed to lose weight, exhibit neurologic symptoms, or succumb to infection (Fig. 2D and E). We confirmed that the avirulent POWV-LI9P-infected mice elicited neutralizing POWV antibody responses (Fig. 3B) and found that mice infected with LI9P were protected from a subsequent lethal LI9 challenge (Fig. 3C and D). Prior LI9P infection prevented spongiform encephalopathy, neurologic sequelae, and microglial activation responses following lethal LI9 challenge. These data suggest that LI9P could serve as a backbone for designing a live-attenuated vaccine.

Prior studies have tested a chimeric YFV-17D/POWV live-attenuated virus as a murine vaccine; however, following two doses, it was only 70% protective, and to reach 100% protection, further immunization with a recombinant EDIII protein was required (43). In contrast, a single LI9P immunization provided 100% protection from a lethal POWV challenge. Other studies have characterized POWV virus-like particles (VLPs) as potential vaccines and observed protective adaptive immune responses in mice (44, 45). However, similar to inactivated TBEV vaccines, the longevity of protective responses from VLPs/subunit vaccines are short lived and likely to require repetitive booster vaccinations (46–49). Although live-attenuated vaccines may have higher risk associated with vaccination of immunocompromised individuals (50), live-attenuated vaccines are highly immunogenic eliciting protective humoral responses and often provide lifelong immunity (31, 51). The ability of passage-attenuated LI9P to protect mice from neuroinvasion and lethality provides rationale for defining determinants of POWV pathogenesis and attenuation.

Previous studies identified EDIII residues 308–311 as targets for TBEV attenuation, and X-ray crystallography-generated structures indicate that TBEV EDIII residue D308 forms a salt bridge with K311 (33). Since EDIII residues 308/311 are conserved in TBEV and POWV (36) and D308N distinguishes attenuated LI9P from LI9, we CPER generated recPOWVs with Env 308/311 residue changes. Like attenuated LI9P, infecting mice with recLI9-D308N failed to cause body weight loss, neurologic symptoms, or lethality (Fig. 4C and D). Remarkably, this indicates that POWV-LI9 was completely attenuated by a single Env protein amino acid change D308N. However, the reciprocal N308D change in recLI9P-N308D was not fully virulent, with a 10% lethality rate that is significantly lower than 80% lethality following LI9 infection (Fig. 4D and E). In addition, a majority of surviving mice infected by LI9P-N308D did not lose weight or show neurologic symptoms, suggesting that in addition to D308N, additional nonstructural protein mutations contribute to LI9P attenuation. Additional analysis of nonstructural protein mutations in LI9P is required to further identify attenuating functions and cell-specific responses that are suggested by differences in LI9 vs LI9P replication and IFN differences (Fig. 7). More attenuating mutations in LI9P are likely to be advantageous for the development of a safe live-attenuated vaccine (49). Further passage of LI9P in VeroE6 cells (10 passages) did not result in reversion of any LI9P residues back to LI9, suggesting the stability of the LI9P virus and its potential as a vaccine or future vaccine framework.

Since recLI9-D308N is fully attenuated, we hypothesized that putative salt bridge interactions stabilize the Env protein and enable binding and infectivity of target cell types. We sought to disrupt this interaction by generating a recPOWV with a mutated K311 residue. Interestingly, replacing K311Q in EDIII generated a viable recombinant virus, but following the insertion of hydrophobic amino acids (K311A or K311F) in EDIII, we were unable to recover viral progeny. These findings demonstrate the permissive selectivity of EDIII 311 residues for POWV viability and led us to assess recLI9-K311Q mutant in mice. In contrast to the complete attenuation of recLI9-D308N, all mice infected with recLI9-K311Q lost weight and displayed neurological symptoms, resulting in partial attenuation of murine lethality (20%). This suggests that Env D308 has a fundamental role in POWV neurovirulence and is only partially dependent on Env K311 or the putative salt bridge interaction. We investigated whether swapping putative salt bridge residues in recLI9-D308K/K311D was attenuating. RecLI9-D308K/K311D replicates slower *in vitro* (Fig. 4B) and to lower viral loads in sera, spleen, and brains, resulting in partial attenuation (90% murine survival) (Fig. 4D). In TBEV studies, a similar inverted salt bridge mutant TBEV-D308K/K311E exhibited decreased replication rates *in vitro* and was partially attenuated *in vivo* (33). However, none of the TBEV mutants described were completely avirulent. Collectively, our findings suggest that changes in the overall charge of EDIII residues may be impacting POWV cellular attachment and potentially targeting POWV to specific cell types that distinguish LI9-D308 neurovirulence from LI9P-N308 attenuation.

Kinetic analysis of POWV RNA viral loads in sera and spleens of infected mice indicated that all POWV strains analyzed are able to replicate systemically, yet POWVs that had higher prolonged viral loads were associated with increased lethality. Regardless of clear differences in lethality between virulent and attenuated POWV strains, similar levels of viremia were detected at 4 dpi. Furthermore, similar splenic viral loads were observed at 4 dpi, but sustained high viral loads in the spleen at 10 dpi are associated with virulent POWVs (LI9). Parallel studies of POWV infected collaborative cross (CC) mice found that susceptibility or resistance to POWV directed lethality is influenced by host factors (52). This study linked sustained high POWV viral loads, consistent with delayed viral clearance, to POWV lethality (52). Collectively, these findings indicate that increased and sustained POWV viral loads are associated with POWV neurovirulence and lethality.

Prior studies of LI9 in 10–50-week-old C57BL/6 mice linked microglial cell activation with POWV pathogenesis (23). The average onset of neurologic symptoms in C57BL/6 mice following LI9 infection is ~10 dpi, which provides a time point during infection to evaluate CNS viral load and microglial activation. In this study, we found that LI9, recLI9P-N308D, and recLI9-K311Q infection of mice resulted in increased CNS viral loads, microglial activation, and increased POWV lethality compared to brains from LI9P, recLI9-D308N, and recLI9-D308K/K311D infected mice. These results further correlate virulent or partially virulent POWVs with the activation of microglia in the CNS and with POWV pathogenesis and lethal outcome.

Since POWV causes viremia, the ability of LI9 to persistently infect hBMECs and release infectious virus both apically and basolaterally provides a plausible mechanism for POWV neuroinvasion. Previous studies of LI9 infection of hBMECs demonstrated that LI9 does not cause decreases in trans-endothelial electrical resistance (TEER); thus, LI9 does not permeabilize hBMEC monolayers *in vitro* (21). Nonetheless, POWV does not need to permeabilize the BBB to enter CNS tissues in this model since it can infect, replicate, and transmit infectious virus bidirectionally from endothelial cells (37–39). Our findings indicate that attenuated LI9P is unable to establish a persistent infection in hBMECs, most likely due to early increases in LI9P RNA synthesis that induce restrictive IFN responses (Fig. 7F through I). LI9 infection of hBMECs causes lower IFN induction at 2–3 dpi compared with LI9P, which delays IFN responses (Fig. 7F through I), potentially enabling the persistent infection in hBMECs by LI9. In contrast, LI9P replication in hBMECs is restricted with decreases in POWV antigen and titers by 3 dpi linked with higher IFN induction and secretion (Fig. 7). Despite being comparably infected (MOI of 1; Fig. 7D), LI9P directs increased RNA synthesis at 1 dpi compared with LI9 (Fig. 7E), and this likely contributes to accelerated IFN induction and secretion, limiting LI9P replication in hBMECs. In YFV studies comparing WT Asibi to vaccine strain 17D, there was an increase in IFN-β and ISG induction following 17D infection compared with WT Asibi (53). Similar to increased LI9P RNA at 1 dpi in hBMECs (Fig. 7E), there were higher amounts of the vaccine strain 17D in the cells compared with WT Asibi, likely contributing to the increased IFN responses (53). Altogether, these data suggest that subversion of IFN responses is important for establishing a persistent POWV infection in hBMECs and may influence POWV neurovirulence.

Flaviviruses must be able to subvert innate immune responses, such as interferon induction, to establish an infection within the host (35, 54–56). Common flavivirus pathogen-associated molecular patterns (PAMPs) include double-stranded RNA (dsRNA) and single-stranded RNA (ssRNA), which can be sensed by pattern recognition receptors (PRRs) and lead to downstream antiviral and interferon responses that consequently limit replication (57, 58). An important flavivirus immune evasion mechanism is the formation of replication complexes, which bends the ER membrane into vesicle packets that shield flavivirus dsRNA intermediates from PRRs (56, 59). Nonstructural proteins aggregate into replication complexes on the ER surface to generate membrane curvature and enable the formation of vesicle packets (59–61). Mutations in nonstructural proteins may alter replication complex formation and result in a change in ER membrane curvature leading to more PAMP recognition by PRRs (62). Consequently, mutations in nonstructural proteins have been shown to be attenuating for many flaviviruses (63–66). For example, a single C100S mutation in NS4B of Zika virus causes increased IFN responses and fully attenuates the strain in AB6 mice (64). Consistent with this, LI9P has six amino acid mutations located in POWV nonstructural proteins (three in NS3, one in each: NS4A, NS4B, and NS5), which may alter virulence. Early RNA replication in LI9P-infected hBMECs along with LI9P nonstructural protein mutations may elicit innate immune responses underlying differences in IFN induction and secretion observed following LI9P versus LI9 infection of hBMECs.

POWV replication is completely restricted by IFN-α pretreatment (21), implying that IFNs may be used as therapeutics. The suggestion that LI9P induces higher IFN expression in hBMECs underlies the potential for restricted LI9P entry into the CNS entry *in vivo*. If LI9P induces heightened or accelerated IFN responses *in vivo*, this could similarly influence immune responses to infection. Type I IFNs have been shown to regulate B-cell induction and the rate of antibody responses (67, 68). Overall, this suggests that more rapid IFN induction could contribute to the protection of LI9P-infected mice.

In this study, we reveal EDIII residue D308 as a primary determinant of POWV neurovirulence and EDIII residues as focal points for POWV attenuation. We demonstrate that sustained high POWV loads, systemically and in the CNS, and the lack of POWV clearance are linked to increased POWV lethality. Most notably, our findings demonstrate that both a recombinant attenuated LI9-D308N virus and a passage-attenuated POWV (LI9P) fail to cause neurologic sequelae or lethality in immunocompetent mice. RecLI9-D308N and LI9P elicit responses that protect mice from lethal POWV challenge and are potential platforms for further POWV vaccine development.

## MATERIALS AND METHODS

### Cells and virus

VeroE6 cells (ATCC CRL 1586) were cultured in Dulbecco’s modified Eagle’s medium (DMEM) at 37°C in 5% CO_2_ as previously described (21, 69). POWV strain LI9 (GenBank accession number: MZ576219) was isolated from field collected *Ixodes scapularis* collected from Long Island, NY. Tick homogenates were inoculated onto Vero E6 cells and passaged 3–5 times in VeroE6 cells to generate stocks (21). Attenuated POWV strain LI9P was generated via infection of VeroE6 cells with LI9 and passaging persistently infected VeroE6 cells 1–2 times per week for ~4 months; LI9P was clonally isolated by end-dilution and subsequently passaged 4–5 times in VeroE6 cells to generate stocks. LI9P was sequenced, compared with LI9, and evaluated for stability and reversion following 10 additional passages.

All work with infectious POWV was performed in approved BSL3 containment facility. POWVs were adsorbed onto 70% confluent VeroE6 cell monolayers for 2 h. Monolayers were washed and grown in DMEM containing 5% FBS. POWV titers were determined by focus assay on VeroE6 cells. Briefly, POWV antigen-positive cells were quantitated after immunostaining cells with anti-POWV hyperimmune mouse ascites fluid (HMAF; 1:5,000; ATCC), secondary horseradish peroxidase (HRP)-labeled anti-mouse IgG (1:2,000; KPL-074-1806), and HRP substrate 3-amino-9-ethyl carbazole (AEC).

Human brain microvascular endothelial cells (hBMECs) were purchased from Cell Biologics (H-6023) grown in EC basal medium-2 MV (EBM-2 MV; Lonza) supplemented with EGM-2 MV SingleQuots (Lonza), and incubated at 37°C and 5% CO_2_. hBMECs were seeded (passages 4–12), and POWVs were adsorbed onto 70% confluent cell monolayers for 2 h, monolayers were washed, and media were replaced. For Transwell experiments, hBMECs were plated on 3 mm pore size Costar Transwell inserts (Corning) and grown for 7 days. Polarization was confirmed using trans-endothelial electrical resistance (TEER) (VOM2, STX3; World Precision Instruments Inc.) prior to POWV infection. Apical and basolateral transwell chamber supernatants were collected at 3 dpi, and titers were determined by focus assay on VeroE6 cells as described above.

### Mice

C57BL/6J mice (10- and 50-week-old) were purchased from Jackson Laboratory. Mice were anesthetized with 100 mg of ketamine and 10 mg of xylazine per kilogram of body weight via intraperitoneal injection. Anesthetized mice were injected subcutaneously in the footpad with DMEM (mock) or 2,000 FFU POWV in a 20 µL volume. Mice were weighed and evaluated daily for signs of clinical neurologic disease (weak grip, ataxia, lethargy, and paralysis). Euthanasia criteria included non-responsiveness or severe neurologic symptoms (hindlimb paralysis, ataxia, and inability to self-right). Alternatively, mice were sacrificed at predetermined time points (4, 10, and 50 dpi) to harvest sera, spleens, or brains for analysis. POWV footpad inoculation and murine lethality studies were replicated in two independent experiments.

### Histopathology

Brains were harvested, fixed in 10% neutral buffered formalin for 7 days, dehydrated with 70% ethanol for 24 h, and paraffin embedded. Formalin-fixed paraffin-embedded (FFPE) brain tissues were sectioned and hematoxylin and eosin (H&E) stained by the SBU Histology Core Lab. Immunohistochemical staining was completed by HistoWiz, Inc., using anti-Iba1 antibody (Wako 019-19741) to identify microglia. Tissue sections were analyzed using QuPath software (https://qupath.github.io). ImageJ was used to quantify percent area of Iba1 + pixel intensity for 10 regions per brain and compared with age-matched mock-infected controls. Spongiform encephalopathic lesions were measured using ImageJ to quantify percent area of the lesion for 10 regions per brain.

### Neutralizing antibody assay

C57BL/6 mice were s.c. footpad inoculated with 2,000 FFU of POWV or mock infected with DMEM. Neutralizing antibodies present in sera from POWV were assessed at 20 dpi using a POWV LI9 focus reduction neutralization assay. Serially diluted sera were incubated with POWV LI9 (500 FFU/well) for 1 h prior to adsorption to VeroE6 cells, and 36 hpi cells infected cell foci were detected by anti-POWV HMAF (1:5,000; ATCC) and immunoperoxidase staining. Neutralizing antibody titers were calculated by quantifying serum dilutions that reduced POWV foci count by 50% (IC50).

### RNA extraction and qRT-PCR

Blood was collected via cardiac puncture, sera isolated (Z-gel microtubes, Avantor), and RNA purified NucleoSpin kit (Macherey-Nagel). Mock or POWV-infected brains were harvested and homogenized in TRIzol LS Reagent (Invitrogen) then RNA purified using Monarch RNA Cleanup Kit (NEB T2030L). Mock or POWV-infected spleens were harvested and homogenized in RLT (QIAGEN) then RNA purified using NucleoSpin kit (Macherey-Nagel). All RNAs were quantified on a Nanodrop Spectrophotometer 2000. To evaluate viral loads in POWV-infected samples, qRT-PCR was performed on cDNAs generated from age-matched mock and POWV-infected sample RNAs. cDNA synthesis was performed using random hexamer priming of a ProtoScript First Strand cDNA Synthesis Kit (New England Biolabs). Viral RNA levels were assayed using LI9-specific primers (Table 1) and compared with a standard curve of serially diluted pLV-POWV-NS5-Flag expression plasmid. Transcript levels were analyzed in triplicate using PerfeCTa SYBR green SuperMix (Quanta Biosciences) on a Bio-Rad C1000 Touch system with a CFX96 optical module.

**TABLE 1:** Primers

Primer	Direction	Sequence
POWV NS5	Forward	GAAACAATACTCAGAATCATG
	Reverse	AAGCCGCTGATCCAGTGGCA
D308N	Forward	GCACAACCTACTCCATGTGTAACAAAAC
	Reverse	GTTTTGTTACACATGGAGTAGGTTGTGC
N308D	Forward	ACTCCATGTGTGACAAAACAAAGTTCAA
	Reverse	TTGAACTTTGTTTTGTCACACATGGAGT
K311Q	Forward	AAAACACAGTTCAAGTGGAAAAGAGTT
	Reverse	AACTCTTTTCCACTTGAACTGTGTTTT
D308K/K311D	Forward	ACAACCTACTCCATGTGTAAAAAAACAGACTTC
	Reverse	GAAGTCTGTTTTTTTACACATGGAGTAGGTTGT
Human IFN-β	Forward	AGTAGGCGACACTGTTCGTG
	Reverse	GAGAAGCACAACAGGAGAGCA
Human IFN-λ	Forward	CGCCTTGGAAGAGTCACTCA
	Reverse	GAAGCCTCAGGTCCCAATTC
Human GAPDH	Forward	AATGAAGGGGTCATTGATGG
	Reverse	AAGGTGAAGGTCGGAGTCAA
Murine IFN-β	Forward	CTGGAGCAGCTGAATGGAAAG
	Reverse	CTTCTCCGTCATCTCCATAGGG
Murine IL-12	Forward	GGAAGCACGGCAGCAGAATC
	Reverse	AACTTGAGGGAGAAGTAGGAATGG
Murine CCL2	Forward	TCAGCCAGATGCAGTTAACG
	Reverse	CTCTCTTGAGCTTGGTGACA
Murine TNF-a	Forward	CGTCGTAGCAAACCACCAAG
	Reverse	TTGAAGAGAACCTGGGAGTAGACA
Murine CXCL10	Forward	AGTGCTGCCGTCATTTTCTG
	Reverse	ATTCTCACTGGCCCGTCAT
Murine IL-6	Forward	TGGGAAATCGTGGAAATGAG
	Reverse	CTCTGAAGGACTCTGGCTTTG
Murine GAPDH	Forward	AATGGTGAAGGTCGGTGTG
	Reverse	GTGGAGTCATACTGGAACATGTA

Mock or POWV-infected VeroE6 cells or hBMECs were lysed using RLT, RNAs purified using RNeasy column (Qiagen), cDNAs generated (New England Biolabs), and qRT-PCR performed. Responses were normalized to internal GAPDH mRNA levels, and the fold induction was calculated using the threshold cycle (2-ΔΔCT) method for differences in RNA levels relative to mock controls at each time point post-infection.

### PCR amplification and CPER reverse genetics

Five LI9 or LI9P fragments were amplified from viral cDNA using high-fidelity Phusion polymerase (NEB) and corresponding paired primers that have a complementary 26-nucleotide overlap as previously described (22). An additional UTR linker fragment was generated from plasmid pMiniT containing the CMV promoter, the first and the last 26 nts of the POWV-LI9 sequence, an HDVr, and SV40 polyadenylation site. POWV cDNA was amplified into six individual POWV DNA fragments: initial denaturation 98°C for 30 s; 32 cycles of 98°C for 20 s, 60°C for 30 s, and 72°C with 30 s per kb; and a final extension at 72°C for 5 min. Amplified fragments were purified using Monarch DNA Gel Extraction kit (New England Biolabs), and 0.1 pmol of each DNA fragment was CPER amplified in a 50 µL reaction containing 200 µM of dNTPs, 1 × Phusion polymerase GC reaction buffer, and 1 µL Phusion polymerase. The following cycling conditions were used: initial denaturation 98°C for 30 s; 12 cycles of 98°C for 20 s, 60°C for 30 s, and 72°C for 10 min; and a final extension at 72°C for 10 min. For generating LI9-D308N, LI9P-N308D, LI9-K311Q, and LI9-D308K/K311D mutant viruses, sub-fragments of fragment F1 were generated by PCR from POWV cDNA, with primers containing codon changes (Table 1) to generate Env proteins containing desired mutations.

### Transfection and recPOWV rescue

CPER reactions were purified (Monarch Gel Extraction Kit, NEB), eluted in sterile H2O, and transfected into VeroE6 cells seeded in 12-well plates using Lipofectamine 3000 reagent (Invitrogen). Supernatants were harvested 2 days post-transfection and amplified in VeroE6 cells to generate viral stocks. Total RNA from VeroE6 cell infections with LI9-D308N, LI9P-N308D, LI9-K311Q, or LI9-D308K/K311D was purified using RNeasy kit (Qiagen). cDNA synthesis was performed as above using random hexamer primers. Mutants were sequenced as described using the SBU Genomics facility.

### ELISA

Secreted IFN-β and IFN-λ in mock and POWV-infected hBMEC supernatants were quantified using DuoSet ELISA (R&D Systems) according to the manufacturer’s instructions. ELISA plates (Immunolon 2, U-bottom; Dynatech Laboratories) were developed using tetramethylbenzidine, and optical density was quantified at 450 nM using Molecular Devices SpectraMax iD3 plate reader. Protein concentrations were calculated by fitting optical density to a standard curve of serially diluted purified IFN-β and IFN-λ.

### Statistical analysis

The statistical significance of results was determined using Prism 10 software (GraphPad Software, Inc.). Statistical analysis for each graph is stated in the corresponding figure legends. IHC quantitation is derived from QuPath and ImageJ analysis of at least 10 brain locations from each of three individual mice. Evaluation of the lethality of recPOWVs in mice has been replicated in two independent experiments. Kaplan–Meier curves were analyzed by a log rank test. Comparisons to mock-infected controls were performed by one or two-way ANOVA. P values of < 0.05 were considered significant.

### Biosafety and biosecurity

Animal studies were conducted in accordance with guidelines outlined in approved protocols that are supervised by the SBU Institutional Biosafety and Institutional Animal Care and Use Committees. Animals were managed by the SBU Division of Laboratory Animal Resources, accredited by the American Association for Accreditation of Laboratory Animal Care and DHHS, and maintained in accordance with the Animal Welfare Act and DHHS “Guide for the Care and Use of Laboratory Animals.” Veterinary care was directed by full-time resident veterinarians accredited by the American College of Laboratory Animal Medicine. POWV murine infection experiments were performed in an animal biosafety level 3 facility (The Laboratory of Comparative Medicine, Stony Brook University).

## Data Availability

The methods underlying this study are available in an online supplement at the authors’ institutional website and by request.

## SUPPLEMENTAL MATERIAL

Fig. S1 mbio.00065-25-s0001.tiff Cytokine CNS responses.

Fig. S2 mbio.00065-25-s0002.tiff LI9-D308N challenge.

Fig. S3 mbio.00065-25-s0003.tiff Mutant HBMEC IFN responses.

ASM does not own the copyrights to Supplemental Material that may be linked to, or accessed through, an article. The authors have granted ASM a non-exclusive, world-wide license to publish the Supplemental Material files. Please contact the corresponding author directly for reuse.
