# Supplementary figures and images for: Passage-attenuated Powassan virus LI9P protects mice from lethal LI9 challenge and links envelope residue D308 to neurovirulence
Source: mBio. 2025 Feb 25;16(4):e00065-25. doi: 10.1128/mbio.00065-25 (PMC11980571; doi:10.1128/mbio.00065-25)

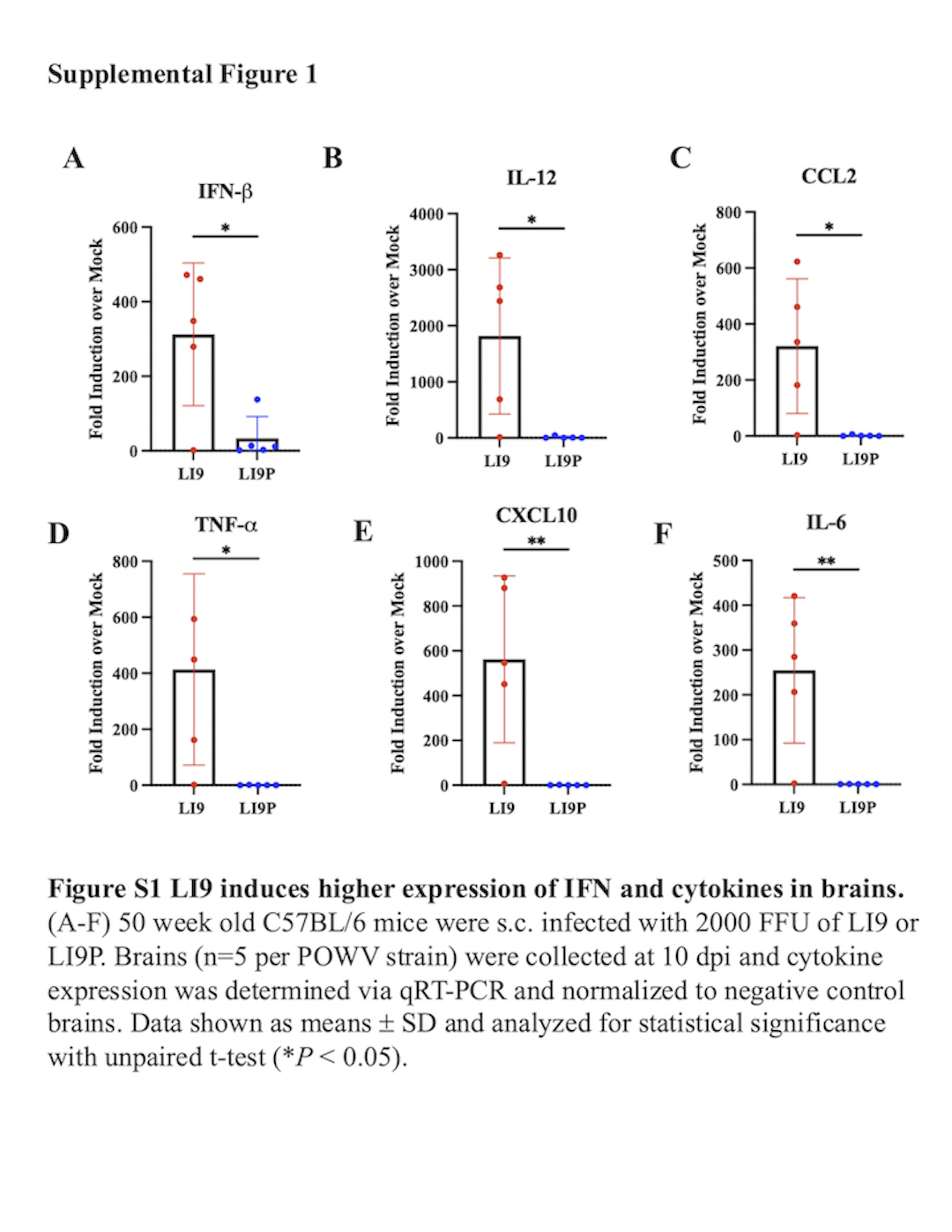

Supplement: Fig. S1 — Cytokine CNS responses. [file mbio.00065-25-s0001.tiff]

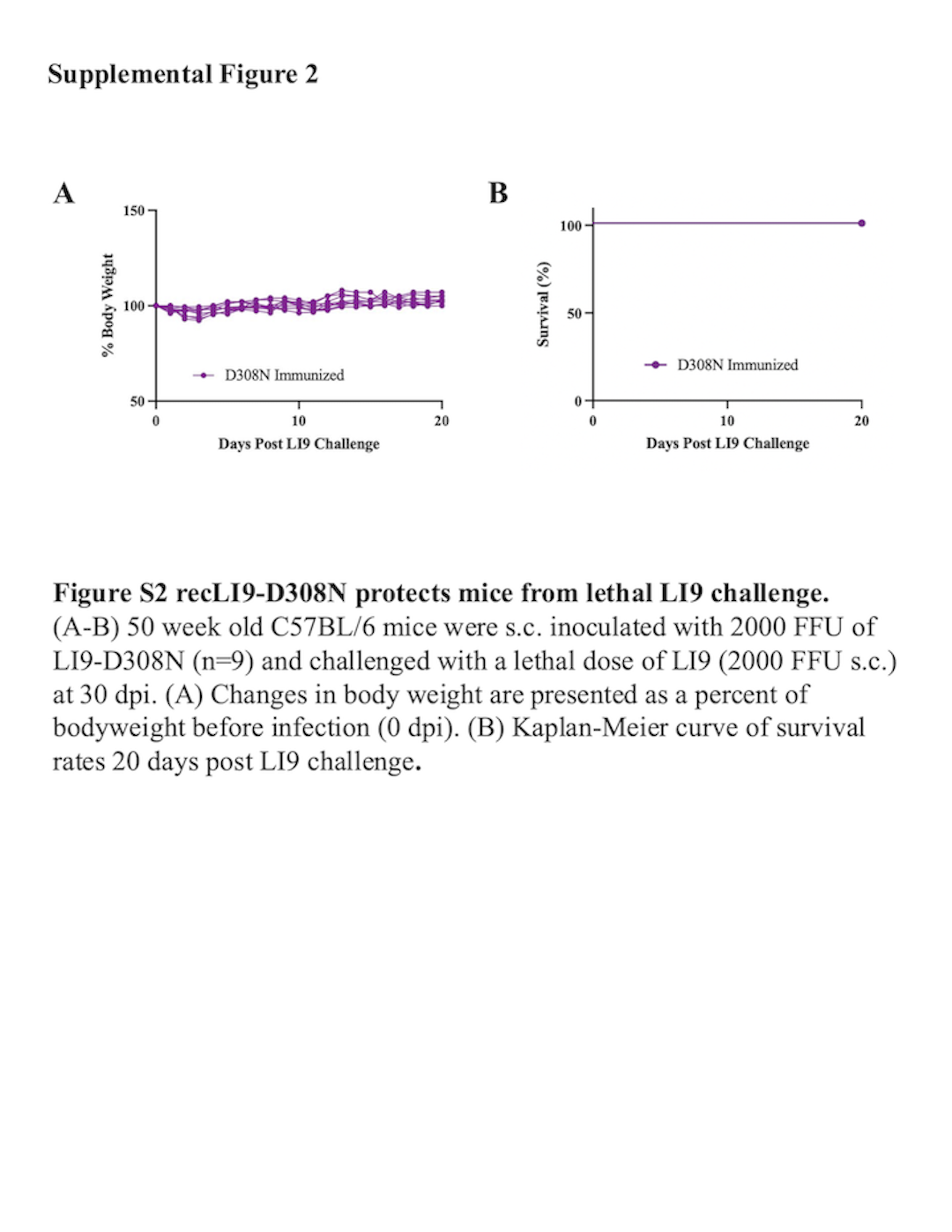

Supplement: Fig. S2 — LI9-D308N challenge. [file mbio.00065-25-s0002.tiff]

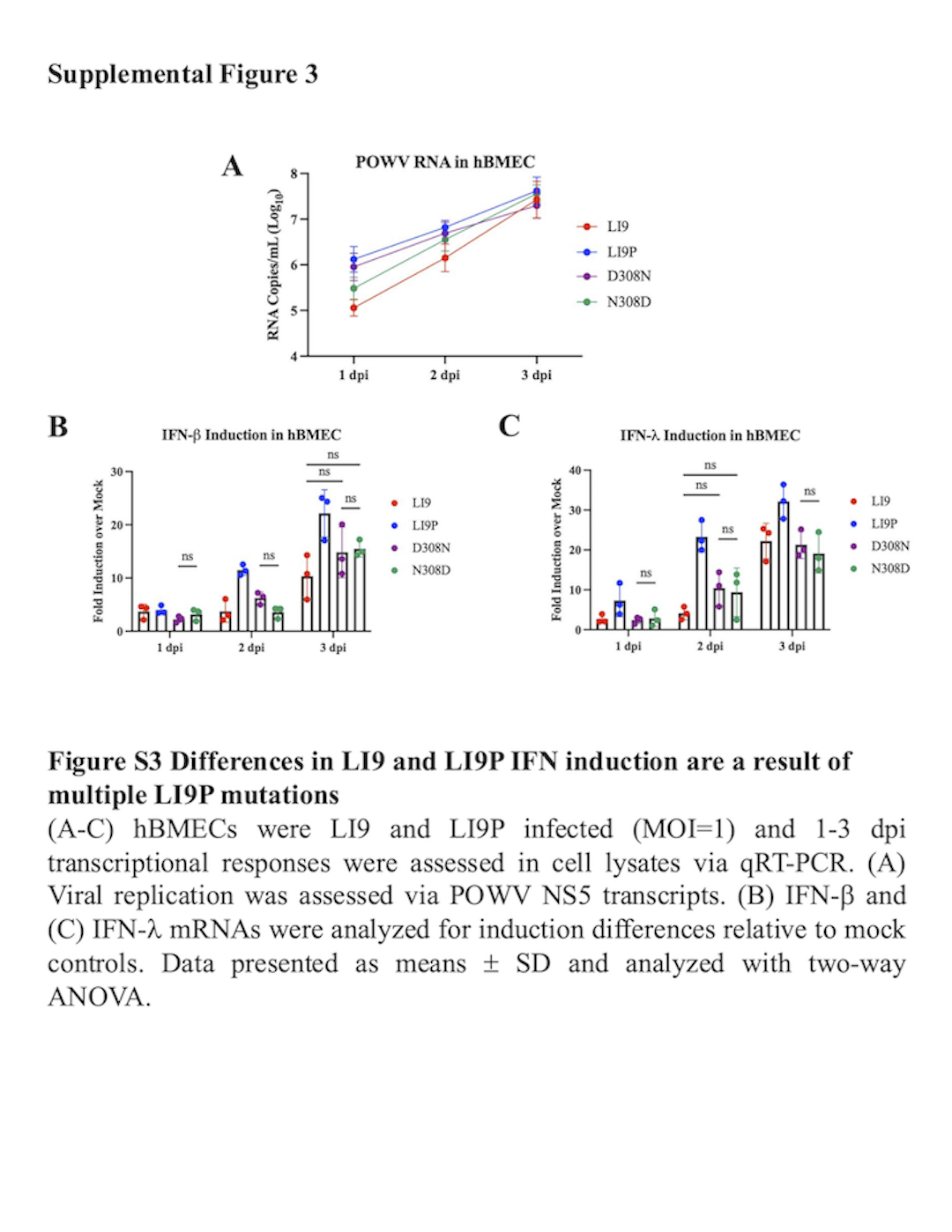

Supplement: Fig. S3 — Mutant HBMEC IFN responses. [file mbio.00065-25-s0003.tiff]
